# Categoricity by convention

**DOI:** 10.1007/s11098-021-01606-3

**Published:** 2021-05-28

**Authors:** Julien Murzi, Brett Topey

**Affiliations:** Philosophy Department (KGW), University of Salzburg, Salzburg, Austria

**Keywords:** Putnam’s model-theoretic argument, Carnap’s Categoricity Problem, Categoricity, Conventionalism, Higher-order logic, Open-ended rules, Permutation invariance

## Abstract

On a widespread naturalist view, the meanings of mathematical terms are determined, and can only be determined, by the way we use mathematical language—in particular, by the basic mathematical principles we’re disposed to accept. But it’s mysterious how this can be so, since, as is well known, minimally strong first-order theories are non-categorical and so are compatible with countless non-isomorphic interpretations. As for second-order theories: though they typically enjoy categoricity results—for instance, Dedekind’s categoricity theorem for second-order PA and Zermelo’s quasi-categoricity theorem for second-order ZFC—these results require full second-order logic. So appealing to these results seems only to push the problem back, since the principles of second-order logic are themselves non-categorical: those principles are compatible with restricted interpretations of the second-order quantifiers on which Dedekind’s and Zermelo’s results are no longer available. In this paper, we provide a naturalist-friendly, non-revisionary solution to an analogous but seemingly more basic problem—Carnap’s Categoricity Problem for propositional and first-order logic—and show that our solution generalizes, giving us full second-order logic and thereby securing the categoricity or quasi-categoricity of second-order mathematical theories. Briefly, the first-order quantifiers have their intended interpretation, we claim, because we’re disposed to follow the quantifier rules in an open-ended way. As we show, given this open-endedness, the interpretation of the quantifiers must be permutation-invariant and so, by a theorem recently proved by Bonnay and Westerståhl, must be the standard interpretation. Analogously for the second-order case: we prove, by generalizing Bonnay and Westerståhl’s theorem, that the permutation invariance of the interpretation of the second-order quantifiers, guaranteed once again by the open-endedness of our inferential dispositions, suffices to yield full second-order logic.

It’s a truism that there’s a close relationship between what linguistic expressions mean and how we use them. In the case of mathematical expressions, at least, these patterns of use amount to inferential roles. A natural metasemantic question to ask, then, is: what, exactly, is the relationship between the inferential roles of mathematical expressions and their meanings?

One possible answer is the crude rationalist answer: we’re in some sort of quasi-perceptual contact with mathematical objects and so can associate linguistic expressions with them in the same way we can associate names with empirical objects; and, just as we make empirical inferences in such a way as to respect what we’ve learned about empirical objects via observation, we make mathematical inferences in such a way as to respect what we’ve learned about mathematical objects via rational insight (see e.g. Gödel 1964). This answer isn’t at all attractive, however: it proceeds by appeal to a supernatural faculty that has no place in contemporary science—the faculty of rational intuition—and so is radically anti-naturalist.

At another extreme is the answer given by radically strong forms of inferentialism: that a mathematical expression’s meaning is simply identical to its inferential role (Putnam 1980; Tennant 1997; Button and Walsh 2018). This view certainly doesn’t proceed by appeal to anything supernatural. But it’s in tension with contemporary formal semantics, which is committed to the thesis that the meaning of an expression is to be understood not in terms of its inferential role but in terms of what it stands for—i.e. of its contribution to the truth conditions of the sentences in which it occurs.^1^

Far more attractive than either of these is the answer given by a *moderate* inferentialism: a mathematical expression’s meaning, on this view, isn’t identical to, but is fully and exclusively *determined* by, our dispositions to infer according to the expression’s basic inference rules. Moderate inferentialism is pre-theoretically plausible, and it also avoids both of the above problems: unlike crude rationalism, it doesn’t require any appeal to the supernatural, and unlike strong inferentialism, it straightforwardly allows us to accept truth-conditional semantics. No surprise, then, that moderate inferentialist views of mathematical language have received wide support, as have similar views of logical language.^2^

This moderate view, attractive as it is, faces a familiar metasemantic challenge: it appears to threaten the very determinacy of our mathematical language. The inferential roles of our mathematical expressions, it seems, can be specified by the principles—axioms and rules—of the theories in which those expressions appear. But if that’s right, indeterminacy looms: it’s an immediate consequence of well-known results in model theory, such as the Löwenheim-Skolem and Compactness theorems, that any (sufficiently powerful) first-order theory is non-categorical—i.e. is interpretable over various non-isomorphic structures—in which case no such theory can on its own single out any one interpretation of our mathematical vocabulary (Skolem 1923; Putnam 1977, 1980, 1981).

To date, no satisfactory (naturalistically respectable) answer to this challenge has been provided. Some have embraced, implausibly in our view, the radical indeterminacy of first-order theories (Skolem 1923; Hamkins 2012). Others have appealed, more plausibly, to second-order logic—i.e. to a logic that allows for quantification not just over objects, but also over sets (or classes) of objects—and to the categoricity (or quasi-categoricity) of second-order mathematical theories—i.e. to the fact that second-order mathematical theories (sometimes with some limitations) have exactly one interpretation, up to isomorphism (see e.g. Shapiro 1991; McGee 1997). However, as it turns out, the principles of second-order logic are themselves non-categorical; to date, no satisfactory metasemantic account has been given of the relation between our use of the second-order quantifiers and their genuine second-order interpretation, on which the categoricity of second-order theories essentially depends. Unless some answer to this challenge is forthcoming, the moderate inferentialist picture of mathematical language is untenable, or so a number of authors have recently argued (Warren and Waxman 2020; Button and Walsh 2018).^3^

In fact, an analogous challenge arises even for moderate inferentialism about *propositional and first-order* logic: as Carnap (1943) famously showed, standard axiomatisations of logic are compatible with non-standard interpretations of negation, disjunction, and the conditional, as well as of the first-order quantifiers—this is Carnap’s *Categoricity Problem*. So, again, there appears to be no way for inferential roles to fix a single interpretation for our logical vocabulary, in which case moderate inferentialism is untenable even for the language of logic (see also Garson 2013).

In this paper, we offer a novel, unified response to these two challenges. Our basic thought is that, although it’s indeed true that the basic principles of our first-order theories can’t fix any single interpretation of our mathematical vocabulary, our dispositions to infer according to the principles of second-order theories, adequately understood, can. We prove three main results. First, *pace* Carnap and Garson, there is available an orthodox, assertion-based, single-conclusion natural deduction calculus for propositional logic whose rules, on an oft-overlooked and yet independently plausible account of validity, uniquely fix, in a naturalistically respectable way, the classical interpretation of the propositional connectives. Second, on the assumption that we accept the rules for the quantifiers in an open-ended way—i.e. that we’re disposed to accept all instances of those rules irrespective of how we expand our language (Harris 1982; McGee 2000)—the interpretation of the quantifiers, both first- and second-order, is guaranteed to be permutation-invariant.^4^ And finally, by a theorem proved by Bonnay and Westerståhl (2016) along with a generalization of that theorem we prove in §2.5, permutation invariance guarantees that the rules for the quantifiers, both first- and second-order, fix those quantifiers’ standard interpretation—in the case of the second-order quantifiers, their so-called *full* interpretation. This is enough to answer the metasemantic challenge for moderate logical inferentialism. But it also paves the way to answering the challenge as it applies to mathematical theories. In the case of arithmetic, for instance, we take our use of the arithmetical vocabulary to be codified by second-order Peano Arithmetic (PA$_{\textsf {2}}$); it’s then an immediate consequence of Dedekind’s categoricity theorem that our dispositions to infer according to basic arithmetical principles uniquely fix, up to isomorphism, the interpretation of arithmetical terms.^5^

To be sure, categoricity arguments depending on the open-endedness of certain schematic principles are not new.^6^ However, existing accounts either focus exclusively on mathematics and so don’t secure the determinacy of our logical vocabulary, or else they depend on provably false assumptions.^7^ Our approach, by contrast, avoids both of these issues. It relies on our moderate inferentialist assumptions (including the open-endedness of logical rules) to prove that our logical expressions (including the second-order quantifiers) meet what, since Tarski (1986), has been widely regarded as at least a necessary condition for logicality, viz. permutation invariance. It then rules out, on this basis, unintended interpretations of the second-order quantifiers. Indeed, our main theorem establishes, quite independently of one’s commitment to any kind of inferentialism, that, insofar as permutation invariance is necessary for logicality, second-order logic *must* have its full interpretation.^8^

The paper is organised as follows. §1 lays out the metasemantic challenge and discusses standard realist responses, finding them wanting. §§2–3 outline our approach and introduce our main results. §4 discusses some potential rejoinders. §5 concludes.

## The metasemantic challenge to realism

According to a standard realist picture, facts about truth are determined by reference relations between linguistic items and bits of the mind-independent world: the sentence ‘Fido is happy’ is true if and only if the referent of ‘Fido’ is in the set of all and only the things to which ‘is happy’ applies, the sentence ‘Someone is happy’ is true if and only if the set of all and only the things to which ‘is happy’ applies is non-empty, and so on.^9^ Realism is most plausibly paired with a *naturalist metasemantics*, a moderate form of inferentialism according to which the meanings of our expressions are exclusively determined by facts about linguistic use.^10^ This sort of naturalism is, we take it, nonnegotiable: what else but the way we use our expressions *could* determine their meanings? Certainly not Gödelian rational insight—this sort of supernatural faculty has no place in contemporary science. In Putnam’s words: ‘either [...] use [...] fixes ‘interpretation’, or nothing can’ (1980, p. 482).

But this naturalist-realist view, compelling as it is, seems to lead to semantic disaster when applied to abstract domains such as mathematics. The problem is that this view seems to force us to accept that the interpretation of our mathematical vocabulary is radically indeterminate. Basic model-theoretic results such as the Compactness and Löwenheim-Skolem theorems show that our best mathematical theories are non-categorical: they’re satisfied by many non-isomorphic structures.^11^ Since there is, and can be, no more to the use of mathematical terms than their occurrence in our best mathematical theories, these results make it a complete mystery how these expressions can have determinate meanings. This is the *Metasemantic Challenge*:

*(MSC)*What, if anything, fixes the interpretation of our mathematical language?

Skolem (1923, p. 296) takes the challenge to be unanswerable—the lesson, for him, is that mathematical notions inevitably fail to have any absolute interpretation. And in a similar vein, Hamkins (2012) suggests that the standard model of arithmetic will eventually cease to be regarded as providing the intended interpretation of arithmetical terms. But others, such as Field (1989), Putnam (1977, 1980, 1981), and Button and Walsh (2018), don’t take the challenge to show that our mathematical notions are indeterminate—while they agree that the MSC is unanswerable on a naturalist-realist view, they take the lesson to be that realism itself is untenable. Button and Walsh, for instance, argue that the realist view is ‘dead’ and introduce in its place a strong inferentialist semantics on which the meanings of mathematical expressions are not merely determined, but *exhausted*, by their inferential roles (2018, Part II).

Both of these options, though, are in our view overly radical. For one thing, the interpretation of our mathematical vocabulary, whatever it is, does *not* seem to be radically indeterminate—this is especially plausible in the case of our arithmetical vocabulary, but our set-theoretic vocabulary seems to enjoy a large degree of determinacy as well. For another, reference-based semantics is a well-established, successful semantic paradigm, accepted by almost all linguists (Williamson 2007, pp. 284–285). While a broadly anti-realist answer to the MSC may in some sense be compatible with this paradigm (Putnam 1980, p. 479), any such answer does put under a great deal of pressure the idea that reference-based semantics ought to be taken at face value.

Our question, then, is whether a satisfying naturalist-realist answer to the challenge is possible. Famously, Putnam’s *model-theoretic argument* (Putnam 1977, 1980, 1981) purports to show that the answer is no—indeed, that metaphysical realism about *any* domain of inquiry makes it impossible to provide a naturalistically respectable account on which the language of that domain has a determinate interpretation.^12^ The basic thought is that it’s guaranteed that any theory we accept, if it’s made true by some interpretation, will be made true by many distinct interpretations, and there’s nothing in our use to pick out one of these interpretations to the exclusion of the others. The key move here—the infamous *just-more-theory manoeuvre*—is to insist that whatever metasemantic account of the intended interpretation of a theory *T* the realist might offer can simply be added to *T*, yielding a stronger theory $T'$ that itself has many distinct interpretations.^13^ If, for instance, a realist about the empirical world maintains, following Putnam (1975) himself and Kripke (1980), that causation fixes reference, Putnam’s response is that adding the sentence ‘causation fixes reference’ to our theory just gives us a new theory, itself up for reinterpretation. Since the relevant model-theoretic results apply to this new theory just as much as to the old one, the new one doesn’t fix reference any more than the old one did, or so Putnam argues.

Realists have forcefully retorted that Putnam’s move here is illicit: what fixes reference, on the view of (for example) the realist about the empirical world, is causation itself, not the *theory* that causation fixes reference.^14^ We think this response is right: if it is indeed correct that causation fixes reference, the fact that the realist’s metasemantic theory can be interpreted in deviant ways is simply irrelevant.^15^ That said, *pace*Bays (2001, 2008), the failure of the just-more-theory manoeuvre isn’t itself an answer to the MSC—some answer still needs to be given. And mathematical realists, unlike realists about the empirical world, can’t appeal to causation here: *ex hypothesi*, mathematical reality is causally inert. But what other strategies are available?

The most common realist move here is to adopt a logic that’s stronger than first-order and then to appeal to various results to the effect that suitably chosen stronger-than-first-order axiomatisations of mathematical theories are categorical.^16^ For instance, if second-order logic (SOL) is available, arithmetic can be axiomatised by means of PA$_{\textsf {2}}$, and realists can then appeal to Dedekind’s well-known categoricity theorem for PA$_{\textsf {2}}$ in order to argue that this axiomatisation fixes the interpretation of our arithmetical vocabulary up to isomorphism.^17^ Related, though weaker, results are available for set theory.^18^ Crucially, though, the results here all require that the second-order quantifiers be interpreted via the so-called *full* semantics for SOL: they must range over the full power set of the *n*-fold Cartesian product of the first-order domain.^19^ This requirement leaves realists’ logic-first approach vulnerable to the objection that it merely moves the underdetermination problem from one place to another.

The worry is that, as is well known, there can be no proof system for SOL that rules out so-called Henkin models on which the second-order quantifiers are restricted, ranging only over some proper subset of the full power set of the *n*-fold Cartesian product of the first-order domain. So it’s difficult to see how our use of language can ever pick out the full interpretation of the higher-order quantifiers to the exclusion of some restricted interpretation.^20^ Indeed, Putnam suggests that realists can answer the question of how the full semantics of SOL is fixed only if they abandon naturalism altogether and ‘attribute to the mind special powers of ‘grasping second-order notions” (Putnam 1980, p. 481). In the case of abstract domains such as mathematics, then, a satisfying, realist-friendly answer to the MSC has not yet been provided: *pace* Bays (2001, p. 350), realists have *a lot* ‘to fear from Putnam and his models’.

That said, we also think that the focus here on whether realists can legitimately appeal to full SOL has led many mathematical anti-realists to fail to take adequate account of a vitally important fact: that the sort of underdetermination problem anti-realists are invoking here for the second-order quantifiers arises *already* for the first-order quantifiers, and even for the propositional connectives. That is, as we know from Carnap (1943), failures of categoricity arise already for propositional and first-order logic, never mind second-order logic, arithmetic, or set theory! Even in the case of the vocabulary of propositional and first-order logic, it’s puzzling how meaning is fixed. In what follows, we argue that the best answer to the question of what determines the intended meanings of the propositional and first-order constants turns out to generalise to the second-order quantifiers as well, allowing a logic-first answer to the MSC by way of Dedekind’s categoricity theorem for second-order arithmetic (and related theorems for second-order set theory).

## Carnap’s Categoricity Problem

Carnap (1943) first showed that standard axiomatisations of classical logic are compatible with non-standard interpretations of the connectives and of the first-order quantifiers. In this section, we introduce, and propose a largely new solution to, Carnap’s Categoricity Problem (§§2.2–2.5). We begin by briefly outlining the moderate inferentialist metasemantic assumptions on which we’re relying (§2.1).

### Moderate inferentialism

We accept a moderate form of inferentialism according to which our dispositions to infer in accordance with basic rules of inference (i) fix the interpretation of our logical and mathematical vocabulary and (ii) constitute our understanding of that vocabulary.^21^ What makes our view moderate is just that, although the meanings of logical and mathematical expressions are fixed by their inferential roles, these meanings are not, on our view, *identical* to inferential roles; rather, the meanings are conceived of in the standard way, as contributions to the truth conditions of the sentences in which the expressions occur (cf. Dummett 1991, p. 205). This moderation is what makes our view compatible with both contemporary formal semantics and orthodox mathematical realism.

For all its moderation, though, our view remains a form of inferentialism. And inferentialism requires, crucially, that our inferential dispositions be syntactic—if our inferential dispositions fix the meanings of our expressions, it certainly can’t be that what explains these dispositions is that we have a prior semantic grasp of the expressions by means of which we recognize that the inferences in question are truth-preserving. Instead, our grasp of these expressions must be *constituted* by our being disposed, as a matter of (implicit) convention, to accept sentences of particular syntactic forms on the basis of our acceptance of sentences of other particular syntactic forms.^22^

The fact that inferential dispositions are syntactic in this way is going to play an important role in our argument for the determinacy of the quantifiers. This fact, after all, makes it plausible that finite creatures like us can have inferential dispositions that are open-ended—i.e. that aren’t sensitive to the language in which the expressions are embedded and so remain undisturbed regardless of how our language is expanded. And the possibility of open-endedness is going to allow us to claim, following (among others) Harris (1982) and McGee (1997, 2000, 2006), that we’re disposed to infer in accordance with the quantifier rules in an open-ended way, in which case those rules remain valid no matter how we expand the language. Before we begin our discussion of the quantifiers, though, we need to ensure that our moderate inferentialist assumptions will allow us to deliver determinacy for at least the propositional connectives.

### Carnap’s Problem I: the connectives

As Carnap (1943, pp. 81ff.) pointed out, standard axiomatisations of classical propositional logic (CPL) are compatible with the following two valuations: the trivial valuation $v^\star$, which makes every sentence of the language true, and the provability valuation $v^\dagger$, which makes every theorem of CPL true and every other sentence false. To see why this is so, let $\vDash$ be the consequence relation of CPL—i.e. the SET–FMLA relation consisting of all the valid arguments of CPL, each represented by an ordered pair of the form $\langle \Gamma , \psi \rangle$, where $\Gamma$ is the set of premises and $\psi$ is the conclusion. It’s easy to see that neither $v^\star$ nor $v^\dagger$ yields counterexamples to the arguments in $\vDash$: on $v^\star$, all sentences are true, in which case there can be no argument in $\vDash$ that has true premises and a false conclusion, and on $v^\dagger$, the only arguments with true premises are those whose premises are theorems of CPL, in which case the conclusion of any propositionally valid argument with true premises will be a theorem of CPL as well. Neither of these valuations, though, is compatible with the standard truth table for the negation sign: on $v^\star$, both $\varphi$ and $\lnot \varphi$ are true, for any $\varphi$, while on $v^\dagger$, both $\varphi$ and $\lnot \varphi$ are false, for any $\varphi$ such that neither $\varphi$ nor $\lnot \varphi$ is a theorem of CPL (e.g. any atomic $\varphi$). Is there any way for moderate inferentialists to maintain, in the face of these results, that the rules of CPL fix the meanings of the propositional connectives?

One strategy we might try here is to beef up our proof-theoretic framework, building more structure into the consequence relation either by adopting a bilateral formalisation of logic on which there are rules governing sentence rejection as well as rules governing sentence acceptance (Smiley 1996; Rumfitt 2000) or by generalising the notion of argument so as to allow arguments with multiple conclusions (Restall 2005). However, while either of these approaches would allow us to rule out Carnap’s non-standard valuations, it’s not entirely obvious that either is compatible with our actual inferential practice, and so both are problematic from an inferentialist perspective.^23^

Bonnay and Westerståhl (2016) have recently proposed a more orthodox approach, one on which CPL is given an assertion-based, single-conclusion formalisation. However, their approach relies (in the case of propositional logic) on two semantic assumptions—to wit, where *v* is an admissible valuation:

Non-triviality According to *v*, the language contains at least one false sentence.

Compositionality For every *n*-ary rule of expression formation $\#$, there’s a semantic composition function $F_\#$ such that, for any well-formed expression $\#(e_1,\dots ,e_n)$, $v(\#(e_1,\dots ,e_n)) = F_\#(v(e_1),\dots ,v(e_n))$.

These assumptions suffice to rule out both of Carnap’s non-standard valuations (the trivial valuation $v^\star$ violates Non-triviality, since it makes every sentence true, while the provability valuation $v^\dagger$ violates Compositionality, since it makes some disjunctions true despite making both disjuncts false—e.g. any disjunction of an atomic sentence with its negation). However, while both of these assumptions are certainly plausible, it’s not obvious that we’re entitled to them in the present context. Inferentialism requires that assumptions about the admissibility of interpretations be justified by appeal to inferential practice, but it’s unclear whether such a justification is available for either Non-triviality or Compositionality.^24^

Garson (2013) points the way toward a more promising strategy, though he doesn’t ultimately endorse it. He explains that whether the validity of the rules of CPL determines the standard meanings for the connectives depends in part on what we take that validity to consist in. Consider, for instance, a formalisation of CPL by means of a single-conclusion natural deduction calculus in sequent style, where, for instance, the basic introduction and elimination rules (henceforth, I- and E-rules) for the conditional are the following metarules: 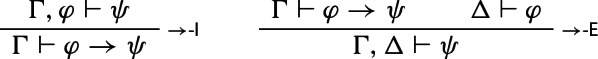


Consider also the notion of *sequent satisfaction*, defined as follows:

#### Definition 1

A sequent $\Gamma \vdash \varphi$ is *satisfied* by a valuation *v* iff either some $\delta \in \Gamma$ is false in *v* or $\varphi$ is true in *v*.

On the usual characterisation of validity, for the rules of our natural deduction calculus to be valid with respect to a class of valuations is just for every sequent provable in the calculus to be satisfied by all the valuations in the class. It is on this characterization that Carnap’s non-standard valuations are not ruled out. But as Garson notes, alternative characterisations are available on which validity requires more than this. In particular, we may characterise the validity of a metarule with premises $\Gamma _1 \vdash \varphi _1, \dots , \Gamma _n \vdash \varphi _n$ and conclusion $\Delta \vdash \psi$ as follows:

#### Definition 2

A metarule is *locally valid* with respect to a class of valuations *V* iff it preserves sequent satisfaction in *V*—i.e. iff for all $v \in V$, if *v* satisfies every $\Gamma _i \vdash \varphi _i$, then it also satisfies $\Delta \vdash \psi$.

Garson (2013, §3.3) shows that the local validity of our rules fixes the standard meanings for the connectives. That is, he proves the following:

#### Theorem 3

The rules of CPL are locally valid with respect to a class of valuations *V* only if all members of *V* obey the classical truth tables.

Unlike Bonnay and Westerståhl’s corresponding result, Garson’s theorem doesn’t rely on Compositionality. It does rely on Non-triviality, but as we show in the next section, this assumption can be eliminated.^25^ Moreover, there’s a case to be made, on inferentialist grounds, that the validity of rules is reasonably characterised as local validity rather than in the usual way. For just as we can accept an argument by finding the move from premises to conclusion compelling—i.e. by being disposed to accept the conclusion conditionally on accepting the premises—so we can also find the move from certain *arguments* to another *argument* compelling. That is, we can be disposed to find one move from premises to conclusion compelling conditionally on finding some *other* moves from premises to conclusion compelling. The usual characterisation of validity can’t capture this conditional sort of argument acceptance, but an account that characterises validity as local validity can: sequents represent arguments, and so a characterisation that allows us to make sense of the validity of rules governing inferences from premise sequents to a conclusion sequent thereby allows us to make sense of the validity of rules governing conditional argument acceptance.

Garson himself rejects the local account of validity, for two main reasons. First, there’s an *incompleteness problem*: by Theorem 3, $\rightarrow$-I and $\rightarrow$-E determine the classical truth table for $\rightarrow$, but there are classically valid principles that involve only that connective but that aren’t derivable using only $\rightarrow$-I and $\rightarrow$-E along with structural rules.^26^ And second, Garson argues that the local account doesn’t generalise properly beyond the propositional case: the quantifier rules, for instance, fail to be locally valid with respect to valuations that respect the standard meanings of the quantifiers. Consider e.g. the standard sequent-style formulation of $\forall$-I: 
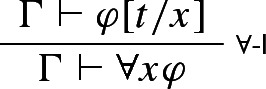
 where *t* occurs neither in $\Gamma$ nor in $\varphi$.^27^ As Garson points out, ‘A valuation that satisfies the argument $\Gamma \vdash \varphi [t/x]$ need not satisfy $\Gamma \vdash \forall x \varphi$, even when the term *t* is missing from $\Gamma$ or $\varphi$’ (2013, p. 43; Garson’s symbolism has been adapted to ours). For instance, $\vdash (t \text { is a city})$ is satisfied if we take *t* to refer to Vienna, but $\vdash \forall x(x \text { is a city})$ certainly is not. Analogous problems arise for $\exists$-E and for modal logic’s necessitation rule.

For these reasons, Garson adopts an alternative account of validity, on which the validity of a rule is characterised in terms of sequent *validity* rather than sequent *satisfaction*. In particular, he characterises validity as *global validity*, where what it is for a metarule with premises $\Gamma _1 \vdash \varphi _1, \dots , \Gamma _n \vdash \varphi _n$ and conclusion $\Delta \vdash \psi$ to be globally valid is as follows:

#### Definition 4

A metarule is *globally valid* with respect to a class of valuations *V* iff it preserves sequent validity in *V*—i.e. iff, if all $v \in V$ satisfy every $\Gamma _i \vdash \varphi _i$, then all $v \in V$ satisfy $\Delta \vdash \psi$.

Garson shows that, on this global account, the validity of the rules fixes the *intuitionistic* meanings for the connectives. Furthermore, this account doesn’t suffer from the incompleteness problem, and it generalises properly beyond the propositional case (2013, Chs. 5–8). However, Garson’s approach is highly revisionary: as classical logicians, we’d prefer to be able to show how the *classical* meanings of logical expressions are determined by their basic I- and E-rules. In what follows, we argue that Garson’s objections to the local account are too quick. A version of the local account turns out to be available that avoids both of Garson’s worries and so allows us to deliver the classical meanings of the connectives.

### Solving Carnap’s Problem for propositional logic: local validity

We begin with the natural deduction framework proposed by Murzi (2020). It’s a single-conclusion framework, with one exception: following Tennant (1999) and Rumfitt (2000), Murzi takes $\bot$ to be, not a propositional constant expressing necessary falsehood, but simply a punctuation mark indicating a logical dead end—i.e. a situation in which *no* reasonable conclusion can be drawn. Formally, then, the consequence relation is a SET–SET relation, but the second element of each ordered pair is always either a singleton or the empty set. In addition, Murzi, following Schroeder-Heister (1984), adopts *higher-order* rules—i.e. rules that allow us to assume and discharge *rules* as well as sentences.^28^ These two refinements, taken together, allow us to formulate $\lnot$-E as a pure rule which only involves negation, and classical *reductio* (CRA) as a structural rule (since $\bot$ is now only a punctuation mark), as follows: 
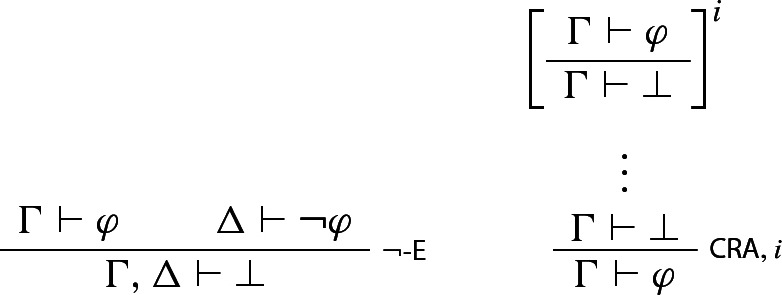
 The reason this is significant is that formulating the rules in this way allows us to avoid Garson’s incompleteness problem completely: Murzi shows that, since CRA on this formulation contains no logical vocabulary and so is a structural rule, any classically valid principle turns out to be derivable using only structural rules along with the I- and E-rules for the connectives that appear in that principle.^29^ And there’s a secondary benefit as well: $\lnot$-E, so formulated, immediately rules out the trivial valuation $v^\star$ and so eliminates the need for us to assume Non-triviality.

Let’s move on, then, to Garson’s second main objection to the local account of validity. We argue that, *pace* Garson, the local account does in fact generalise properly beyond the propositional case. Consider again $\forall$-I, as formulated above. The thing to notice here is that, in any case in which an instance of that rule appears as part of a correct proof in first-order logic (FOL), the premise $\Gamma \vdash \varphi [t/x]$ will have been *proved*. Garson’s alleged counterexamples to the local validity of $\forall$-I ignore this fact, but it’s crucial. What guarantees that uses of the rule in correct proofs won’t lead us into error is precisely that, when the sequent in question is provable, *t* is entirely arbitrary: given the way the rules are set up, if $\Gamma \vdash \varphi [t/x]$ is provable (where *t* doesn’t appear in $\Gamma$ or $\varphi$), then $\Gamma \vdash \varphi [t^\star /x]$ is provable as well, for any $t^\star$. This might suggest that we should simply build an explicit side condition into $\forall$-I that requires that the premise sequent be provable in FOL.

Such a side condition would certainly allow us to avoid Garson’s counterexamples to the local account: any counterexample must be such that $\Gamma \vdash \varphi [t/x]$ is satisfied while $\Gamma \vdash \forall x \varphi$ is not, but in that case $\Gamma \vdash \varphi [t/x]$ can’t be provable. However, building in such a side condition would be overly restrictive: it would disallow many perfectly reasonable uses of $\forall$-I. Suppose that a thinker has some non-logical reason for accepting $\varphi [t/x]$ on the basis of $\Gamma$ (where *t* doesn’t occur in $\Gamma$) and that this reason is entirely insensitive to the semantic value of *t*. Arguably, this thinker can apply $\forall$-I in order to come to accept $\forall x \varphi$ on the basis of $\Gamma$ as well, even if $\Gamma \vdash \varphi [t/x]$ isn’t provable in FOL. So we shouldn’t try to guarantee *t*’s arbitrariness by building a provability side condition into $\forall$-I. Similarly for $\exists$-E. (Note, though, that we *can* use this approach to solve the analogous problem for modal logic’s necessitation rule—that rule already includes a side condition requiring the provability of the premise.)

Still, though, some guarantee of *t*’s arbitrariness is needed—the entire point of requiring that *t* not occur in $\Gamma$ is to ensure that, when we accept $\varphi [t/x]$ on the basis of $\Gamma$, our reason for doing so has nothing to do with *t* itself. That is, the point is to respect the fact that *t*, though formally a referring term, is playing the role of a *variable* in our reasoning. We suggest that we can capture this fact directly, just by adopting a different (though also standard) version of $\forall$-I—one on which what’s playing the relevant role in our reasoning is an actual variable rather than a referring term standing in for a variable: 
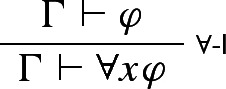


where *x* doesn’t appear free in $\Gamma$. This version of the rule, unlike the previous one, doesn’t allow us to replace a term *t* with a bound variable *x* when we move from premise to conclusion; instead, it allows us, in the course of our reasoning, to use a premise sequent in which *x* appears free.

Now, Garson’s objection, recall, was that $\forall$-I fails to be locally valid with respect to valuations that respect the standard meanings of the quantifiers—$\vdash (t \text { is a city})$, for instance, is satisfied if we take *t* to refer to Vienna, but $\vdash \forall x (x \text { is a city})$ is not. However, given our new formulation of $\forall$-I, the inference from $\vdash (t \text { is a city})$ to $\vdash \forall x (x \text { is a city})$ isn’t an instance of the rule. (The inference from $\vdash (t \text { is a city})$ to $\vdash \forall x (t \text { is a city})$ *is* an instance. But obviously it doesn’t yield a counterexample to the local account of validity, since the use of the quantifier is vacuous.) Garson’s objection, if it is to apply to our new formulation, must be that $\vdash (x \text { is a city})$ can be satisfied despite the fact that $\vdash \forall x (x \text { is a city})$ isn’t—or, more generally, that ‘a valuation that satisfies the argument $\Gamma \vdash \varphi$ need not satisfy $\Gamma \vdash \forall x \varphi$, even when *x* is missing from $\Gamma$’. This version of the objection, though, rests on a mistaken understanding of what it is for a valuation to satisfy a sequent that contains a free variable. To see this, we first note that, since we now allow open formulas to occur in sequents, we must generalise our definition of sequent satisfaction. We do so by first defining a notion of sequent satisfaction relative to a variable assignment:

#### Definition 5

If *s* is a variable assignment, a valuation *v* *satisfies*$_s$ $\Gamma \vdash \varphi$ iff, in *v*, either *s* fails to make true some $\delta \in \Gamma$ or *s* makes true $\varphi$.

We can now use this new notion to generalise our definition of sequent satisfaction, as follows:

#### Definition 6

A sequent $\Gamma \vdash \varphi$ is *satisfied* by a valuation *v* iff *v* satisfies$_s$ $\Gamma \vdash \varphi$ for every variable assignment *s*.^30^

And finally, we define the notion of local validity as before, though now in terms of our generalised notion of sequent satisfaction:

#### Definition 7

A metarule is *locally valid* with respect to a class of valuations *V* iff it preserves sequent satisfaction in *V*.

Again, Garson’s objection, in this setting, is that (e.g.) $\vdash (x \text { is a city})$ can be satisfied even though $\vdash \forall x (x \text { is a city})$ isn’t. Given our new definitions, though, this claim is clearly false, for $\vdash (x \text { is a city})$ is certainly *not* satisfied: it’s satisfied$_s$ if *s* assigns Vienna to *x*, but not if *s* assigns the number seven to *x*. On our new formulation of $\forall$-I, then, Garson’s counterexamples to the local validity of that rule just don’t arise. Similar considerations apply to $\exists$-E.

### Carnap’s Problem II: the first-order quantifiers

So far, so good—our approach delivers determinacy for the propositional connectives, and it isn’t vulnerable to either of Garson’s objections to the local account of validity. But can we deliver determinacy for the first-order quantifiers? As it happens, Carnap’s Problem appears to be even worse in this case: the resources we’ve been relying on up to this point, though they do allow us to explain how use fixes the intended meanings of the connectives, don’t allow us to do the same for the quantifiers. The reason, as we’ll see, is that, even if validity is local validity, the validity of the quantifier rules fails to guarantee that the quantifiers get their standard unrestricted interpretation as opposed to an interpretation on which the language’s variables range only over some proper subset of the domain.^31^ What’s needed is a way to rule out such restricted interpretations.

McGee (2000) and Bonnay and Westerståhl (2016) approach this problem in similar but distinct ways. McGee argues that logical rules are to be understood as open-ended—i.e. that they’re to be understood as valid not just in the language as it currently stands but in all possible expansions of that language—and that the quantifier rules, if understood as open-ended, do determine the unrestricted interpretation. And Bonnay and Westerståhl assume that the interpretation of the first-order quantifiers is *permutation-invariant*—i.e. that it doesn’t change regardless of how the domain of discourse is permuted—and prove, on this assumption, that restricted interpretations are ruled out. As we’ll see, both McGee’s and Bonnay and Westerståhl’s approaches are problematic. McGee’s argument relies, in an essential way, on a demonstrably false assumption. And as for Bonnay and Westerståhl’s assumption of permutation invariance, it simply invites the metasemantic question of what in our use of language makes it the case that the interpretation of the quantifiers must be permutation-invariant. Nonetheless, we think both of these approaches are on the right track.

#### McGee’s approach

McGee (2000) adopts a general strategy for securing logical determinacy that’s quite different from the one we’ve been developing. Rather than understand validity as local validity or set up the proof-theoretic framework in such a way that CRA is a structural rule, he takes open-endedness to be able to do all the necessary work here: he argues that, if logical rules are open-ended, the rules of classical logic uniquely determine the standard interpretation of the logical vocabulary, in both the propositional and the first-order case (and, indeed, in the second-order case as well). We won’t rehearse his reasoning, but we note that he makes the following assumption about what sorts of expansions of a given language are possible:

*McGee’s Principle* For any class of valuations with respect to which our logical rules are valid, there’s an expansion of the language containing a sentence true in exactly those valuations.

McGee relies on an instance of this principle in his proposed derivation of the standard truth conditions for universally quantified sentences—he makes explicit use of the premise that ‘if a class of models is closed under *c*-variants, then there is, in some mathematically permissible language, a sentence not containing *c* that is true in all and only the members of the class’ (2000, p. 71). The problem is that, as Brîncuş (2019) shows, McGee’s Principle is simply false: even in the case of CPL, there are counterexamples.^32^ So, since the principle isn’t true in general, it’s not clear what reason we have to accept this instance of it. An alternative approach to securing first-order determinacy is needed.

#### The First-Order Thesis

What we need to show is that, given our local account of validity, the validity of the rules of FOL guarantees the standard interpretation of the language of FOL. We’ve already argued that the standard interpretation of the propositional connectives is guaranteed; what’s left, then, is showing the same for the quantifiers. That is, we need to prove the following:

First-Order Thesis The rules of FOL are locally valid with respect to a class of valuations *V* only if all $v \in V$ obey the standard interpretation of $\forall$—i.e. are such that, for any $\varphi$ with at most *x* free, $\forall x \varphi$ is true in *v* iff every object in the domain is in the extension of $\varphi$ in *v* (or, more briefly: iff $\mathsf {Ext}_v(\varphi ) = M$, where *M* is *v*’s domain).^33^

With the resources we’ve been relying on up to this point, though, we can’t prove this thesis. The problem, briefly, is that the local validity of quantifier rules doesn’t allow us to rule out the possibility that the variables of our language range only over a proper subset of the domain—i.e. that there’s some object in the domain that no variable assignment ever assigns to a variable. If there’s such an object, *v* can satisfy$_s$ $\vdash \varphi$ for any *s* even if that object isn’t in the extension of $\varphi$, in which case the local validity of $\forall$-I will guarantee that $\forall x \varphi$ is true in *v* despite there being an object that’s not in the extension of $\varphi$.^34^

What we *can* prove is the following weakened version of our thesis:

Weakened First-Order Thesis The rules of FOL are locally valid with respect to a class of valuations *V* only if all $v \in V$ are such that, for any $\varphi$, $\forall x \varphi$ is true in *v* iff $M_x \subseteq \mathsf {Ext}_v(\varphi )$, where $M_x$ is the range of *x* in *v*.

##### Proof

Suppose the first-order rules are satisfaction-preserving in *v*, and let $\varphi$ be any formula with at most *x* free. First, suppose every object in the range of *x* in *v* is in $\mathsf {Ext}_v(\varphi )$. Then *v* satisfies$_s$ $\vdash \varphi$ for any *s*, in which case *v* satisfies $\vdash \varphi$. So, since $\forall$-I is satisfaction-preserving, *v* satisfies $\vdash \forall x \varphi$ as well—i.e. $\forall x \varphi$ is true in *v*. Next, suppose that $\forall x \varphi$ is true in *v*—i.e. that *v* satisfies $\vdash \forall x \varphi$. Then, since $\forall$-E is satisfaction-preserving, *v* satisfies $\vdash \varphi$. So *v* satisfies$_s$ $\vdash \varphi$ for any *s*, which means that every object in the range of *x* in *v* is in $\mathsf {Ext}_v(\varphi )$. $\square$

Local validity guarantees, then, that the range of $\forall$—i.e. the set of objects such that, for any $\varphi$ with at most *x* free, $\forall x \varphi$ is true in *v* iff every object in that set is in $\mathsf {Ext}_v(\varphi )$—is a subset of the domain (in particular, the subset containing exactly those objects over which the variables range). But how do we get from here to the full First-Order Thesis?

#### Bonnay and Westerståhl’s results

We get our answer from Bonnay and Westerståhl (2016), who essentially prove the following theorem:

##### Theorem 8

If the interpretation of $\forall$ is permutation-invariant in some valuation *v* with respect to which the rules of FOL are valid—i.e. if the interpretation of $\forall$ remains the same under all permutations of the domain *M* of *v*—then, if the range of $\forall$ is some subset of *M*, that range must be *M* itself.

It is, of course, an immediate consequence of this theorem that, assuming permutation invariance is guaranteed, the Weakened First-Order Thesis entails the First-Order Thesis. Understanding exactly what’s going on here, though, will require some discussion of the details of Bonnay and Westerståhl’s approach.

On the semantic assumptions Bonnay and Westerståhl allow themselves—including, again, Compositionality—it turns out that, where $\mathcal {L}$ is a first-order language, a model of FOL (i.e. a valuation with respect to which the rules of FOL are valid) can be represented by a pair $\langle \mathcal {M}, Q \rangle$, where $\mathcal {M}$ is an $\mathcal {L}$-structure based on domain *M* and *Q* is a set of subsets of *M* such that, for any $\varphi$ with at most *x* free, $\forall x \varphi$ is true in the model just in case the extension of $\varphi$ is a member of *Q*. The satisfaction clause for $\forall$ is then guaranteed to be the following:

$$\begin{aligned} \mathcal {M}, Q \vDash \forall x \phi \text { iff }\mathsf {Ext}_{\mathcal {M}, Q}(\varphi ) \in Q \end{aligned}$$

To guarantee the standard interpretation for $\forall$, Bonnay and Westerståhl must prove that *Q* is a singleton whose only member is the domain *M*. But they can’t prove this without additional assumptions. What they do prove, though, is that *Q* is a principal filter on *M*—i.e. that, for some $A \subseteq M$, *Q* consists of all and only those subsets of *M* that are supersets of *A*. So the above satisfaction clause reduces to the following:

$$\begin{aligned} \mathcal {M}, Q \vDash \forall x \phi \text { iff }A \subseteq \mathsf {Ext}_{\mathcal {M}, Q}(\varphi ) \end{aligned}$$

where *A* is some subset of *M*. That is, $\forall$ ranges over some $A \subseteq M$. And this, notice, is an immediate consequence of our Weakened First-Order Thesis. In short, although our approach doesn’t rely on semantic assumptions and so is naturalist-friendly in a way that Bonnay and Westerståhl’s approach is not, we’ve managed to arrived at the same result at which they’ve arrived.

Having come this far, we can now go even further: we can accept Theorem 8 on the basis of Bonnay and Westerståhl’s own reasoning, since they don’t here rely on their semantic assumptions. Their reasoning can be sketched as follows. Notice first that, given the satisfaction clauses above, the permutation invariance of the interpretation of $\forall$ in a model of FOL amounts to the permutation invariance of the *range* of $\forall$—i.e. the permutation invariance of *A* or, equivalently, the permutation invariance of *Q*. Bonnay and Westerståhl, then, prove the following set-theoretic lemma:

##### Lemma 9

If a principal filter *F* on *S* is permutation-invariant, $F = \{S\}$.

So $Q = \{M\}$, which means $A = M$. That is, permutation invariance guarantees that, insofar as $\forall$ ranges over some subset of the domain, it ranges over the domain itself. So, if we can assume permutation invariance, we can use our Weakened First-Order Thesis to prove the First-Order Thesis and so can guarantee the unrestricted interpretation of $\forall$. The question arises, though, whether we *can* assume permutation invariance, given our naturalism.

#### From open-endedness to permutation invariance

Bonnay and Westerståhl’s own reason for assuming permutation invariance is that, as almost everyone agrees, it’s a necessary condition for logicality (2016, pp. 725–726): the logical laws should hold universally, in a topic-neutral way, irrespective of what objects one is reasoning about (see also e.g. Tarski 1986). However, if the appeal here to the topic neutrality of logic is to be naturalistically respectable, we must be able to justify, by reference to our inferential dispositions, the claim that our logical expressions are topic-neutral.

To this end, note first that the disposition to infer in accordance with a given logical rule is highly general—we’re disposed to accept any instance of that rule whatsoever, including novel instances. Indeed, we plausibly are disposed to accept even instances involving expressions that aren’t currently in our language: since, as McGee points out, the grounds on which we accept a logical rule in the first place are not that ‘we have surveyed the forms of expression found in English and found that its expressive power is circumscribed in such a way as to validate the rule’, we can continue to rely on that rule even after we have introduced (e.g.) ‘a new predicate into the language’, without having to wonder whether the rule ‘remains valid for inferences involving the new predicate’ (2000, p. 66). That is, we plausibly are disposed to infer in accordance with logical rules in an open-ended way. As discussed above, what makes it plausible that this can be the case, despite the fact that it entails that we’re disposed to accept infinitely many instances of a given rule, is that, given our inferentialism, the dispositions in question must be purely syntactic—we must be disposed to apply a rule not on the basis of any prior grasp of the premises’ meanings but just on the basis of their syntactic forms. Given the syntactic nature of these dispositions, we need to posit only a very simple, finitely specifiable dispositional structure in order to make sense of our being disposed to accept *all* of the infinitely many instances of our logical rules.

Now, given that the meanings of our logical expressions are fixed by these sorts of open-ended dispositions to infer in accordance with logical rules, the rules themselves must be open-ended, in the sense that they remain valid irrespective of how the language is expanded. This fact, together with the highly plausible assumption that no item is in principle unnameable in any language that expands our own, guarantees that the logical rules hold universally: for any item whatsoever, there exists an expansion of our language in which there’s a term naming that item, and the logical rules hold in all such expansions. In fact, given that everything is in principle nameable, the following can be *proved*:

##### Theorem 10

If $\forall$-E is open-ended, then the interpretation of $\forall$ is permutation-invariant in any model of FOL.

We start by making precise our assumption, which is a version of the thesis Pedersen and Rossberg (2010, p. 333) call *McGee’s Rule*. The thesis can be stated as follows:

*McGee’s Rule* Given any model *v* of our language $\mathcal {L}$, the following hold:

1. For any object *o* in the domain of *v*, there’s some language expanding $\mathcal {L}$ in which there’s a term whose referent is *o*.
2. For any collection *C* countenanced by *v*, there’s some language expanding $\mathcal {L}$ in which there’s an open formula whose extension is *C*.

To clarify, we aren’t understanding this as a metasemantic thesis to the effect that the relevant expansions of $\mathcal {L}$ are languages we have any feasible way of coming to speak. That is, our argument doesn’t require the assumption that we have some feasible way of adding new terms to our language so that these terms will determinately name any items we like; to make that assumption would be to assume, question-beggingly, that a solution to the underdetermination problem is available. What we need to assume is only that each of these expansions is a language—i.e. an ‘abstract semantic system[] whereby symbols are associated with aspects of the world’ (Lewis 1970, p. 19)—that *exists*. As we argue more fully in §4, the open-ended structure of our inferential dispositions takes care of the rest.^35^ So McGee’s Rule, as we’re using it, is only a thesis about what sorts of languages are *possible*. (Notice also that, since McGee’s Rule is only about the nameability of items, it’s much weaker than McGee’s Principle as formulated in §2.4.1.) Here’s the proof:

##### Proof of Theorem 10

We prove the contrapositive. Suppose the interpretation of $\forall$ is *not* permutation-invariant in some model *v* of FOL. Then there’s some object not in the range of $\forall$. Let some new term *c* name this object—we know that this is possible, by clause 1 of McGee’s Rule. Then $\forall x \lnot (x = c)$ is true in *v*, and so $\forall$-E can no longer be valid: it would allow us to infer $\lnot (c = c)$, which of course can’t be true in *v* (cf. McGee 2006, p. 187). So $\forall$-E isn’t open-ended.^36^$\square$

With this naturalist-friendly justification of our assumption of permutation invariance, we’ve completed our task of showing, without appeal to contentious semantic assumptions, that the First-Order Thesis is true—i.e. that the rules of FOL determine the standard (unrestricted) interpretation of $\forall$.

### Generalising: the categoricity of higher-order logic

As it turns out, we can generalise Bonnay and Westerståhl’s results to SOL. That is, by reasoning broadly parallel to that just rehearsed, we can show that the following thesis is true.

*Second-Order Thesis* The rules of SOL are locally valid with respect to a class of valuations *V* only if all $v \in V$ obey the standard interpretation of $\forall _2$.

Note first that, just as in the first-order case, it’s possible, without relying on permutation invariance, to prove the following weakened version of this thesis:

Weakened Second-Order Thesis The rules of SOL are locally valid with respect to a class of valuations *V* only if all $v \in V$ are such that, for any $\varphi$, $\forall _2 X \varphi$ is true in *v* iff $M_X \subseteq \mathsf {Ext}_v(\varphi )$, where $M_X$ is the range of *X* in *v*.

#### Proof

Suppose the second-order rules are satisfaction-preserving in *v*, and let $\varphi$ be any formula with at most *X* free. First, suppose everything in the range of *X* in *v* is in $\mathsf {Ext}_v(\varphi )$. Then *v* satisfies$_s$ $\vdash \varphi$ for any *s*, in which case *v* satisfies $\vdash \varphi$. So, since $\forall _2$-I is satisfaction-preserving, *v* satisfies $\vdash \forall _2 X \varphi$ as well—i.e. $\forall _2 X \varphi$ is true in *v*. Next, suppose that $\forall _2 X \varphi$ is true in *v*—i.e. that *v* satisfies $\vdash \forall _2 X \varphi$. Then, since $\forall$-E is satisfaction-preserving, *v* satisfies $\vdash \varphi$. So *v* satisfies$_s$ $\vdash \varphi$ for any *s*, which means that everything in the range of *X* in *v* is in $\mathsf {Ext}_v(\varphi )$. $\square$

By this weakened thesis, local validity guarantees that $\forall _2$ is correctly interpreted via some Henkin semantics—i.e. that, when $\forall _2$ binds some relation variable of arity *n*, it ranges over some subset of the *n*-ary relations. In Bonnay and Westerståhl’s preferred terminology, it’s guaranteed that, where $\mathcal {L}_2$ is a second-order language, a model of SOL can be represented by a pair $\langle \mathcal {M}, Q \rangle$, where $\mathcal {M}$ is an $\mathcal {L}_2$-structure based on domain *M* and *Q* is a set that contains, for each arity *n*, some set $Q^n$ such that $Q^n$ is a principal filter on $\mathcal {P}(M^n)$, the power set of the *n*-fold Cartesian product of *M*. The satisfaction clause for $\forall _2$ is then guaranteed to be the following:

$$\begin{aligned} \mathcal {M}, Q \vDash \forall _2 X \varphi \text { iff }\mathsf {Ext}_{\mathcal {M}, Q}(\varphi ) \in Q^n \end{aligned}$$

where *X* is a relation variable of arity *n*. To put it another way: $\forall _2$, when it binds a variable of arity *n*, is guaranteed to range over some subset of $\mathcal {P}(M^n)$.

This means that, in order to guarantee the full interpretation of $\forall _2$ we must show only that $Q^n = \{\mathcal {P}(M^n)\}$, so that the range of $\forall _2$ is $\mathcal {P}(M^n)$ itself—i.e. the set of all *n*-ary relations on the domain. An application of Lemma 9 tells us that this much is guaranteed by the permutation invariance of the interpretation of $\forall _2$ in our model, just as in the first-order case.^37^ That is, given Lemma 9, the Weakened Second-Order Thesis immediately entails the second-order analogue of Theorem 8:

#### Theorem 11

If the interpretation of $\forall _2$ is permutation-invariant in some model of SOL, then, if the range of $\forall _2$ is some subset of $\mathcal {P}(M^n)$, that range must be $\mathcal {P}(M^n)$ itself.

Furthermore, we know that the open-endedness of $\forall _2$-E guarantees permutation invariance, for we can prove the following second-order analogue of Theorem 10:

#### Theorem 12

If $\forall _2$-E is open-ended, then the interpretation of $\forall _2$ is permutation-invariant in any model of SOL.

#### Proof

As before, we prove the contrapositive. Suppose the interpretation of $\forall _2$ is *not* permutation-invariant in some model *v* of SOL. Then there’s some *n*-ary relation not in the range of $\forall _2$. Let some new term *C* name this relation—we know that this is possible, by clause 2 of McGee’s Rule. Then $\forall _2 X \lnot \forall x_1 \dots \forall x_n (X(x_1,\dots ,x_n) \leftrightarrow C(x_1,\dots ,x_n))$ is true in *v*, and so $\forall _2$-E can no longer be valid: it would allow us to infer $\lnot \forall x_1 \dots \forall x_n(C(x_1,\dots ,x_n) \leftrightarrow C(x_1,\dots ,x_n))$, which of course can’t be true in *v*. So $\forall _2$-E isn’t open-ended. $\square$

So, just as in the first-order case, we’ve shown, on naturalist-friendly grounds, that the Second-Order Thesis is true—i.e. that the (open-ended) rules of SOL fix the full interpretation of $\forall _2$. What’s more, it seems clear enough that our results here will generalise further, to quantifiers of arbitrary (finite) order: our strategy for solving Carnap’s Categoricity Problem generalises to our second-order vocabulary and beyond. Finally, it’s worth stressing that our result is significant independently of one’s commitment to any form of inferentialism: Theorem 11 tells us that, insofar as permutation invariance is a necessary condition for logicality, and insofar as the second-order quantifiers are genuinely logical, the rules for the second-order quantifiers are simply incompatible with any restricted interpretation (and analogously for Theorem 8 and the first-order quantifiers).^38^

## Mathematical categoricity by convention

Let’s take stock: we’ve argued, on naturalist-friendly grounds, that the rules of SOL determine that SOL is correctly interpreted by the full semantics. If this is right, then it turns out that the sort of logic-first answer to the MSC we discussed in §1 is available to mathematical realists, despite Putnamian worries: as we’ve mentioned, full SOL makes available categorical and quasi-categorical axiomatisations of, respectively, arithmetic and set theory. Arithmetic, for instance, can be axiomatised by means of PA$_{\textsf {2}}$—essentially, first-order Peano Arithmetic with the induction schema replaced by the following second-order *axiom*:

.

And as we’ve already noted, Dedekind’s categoricity theorem guarantees that, given full SOL, the axioms of PA$_{\textsf {2}}$ fix, up to isomorphism, the interpretation of the arithmetical vocabulary. So, insofar as the rules of SOL do indeed fix the full semantics, our acceptance of PA$_{\textsf {2}}$ yields the categoricity of arithmetic. Analogously for the quasi-categoricity of set theory, via Zermelo’s quasi-categoricity theorem.^39^

If we’re right, mathematical truths are true by convention, in an attenuated sense that’s consistent with realism. In the case of arithmetic, for instance, our conventions—our dispositions to accept the axioms of PA$_{\textsf {2}}$ and to reason in accordance with the open-ended rules of SOL—determine the interpretation of the arithmetical vocabulary. A standard Tarskian account of truth of the sort discussed in §1 then does the rest, settling the truth value of every arithmetical sentence. Thus, our conventionalism is far less radical than orthodox forms of conventionalism, which typically identify mathematical conventions with principles such as the axioms of Peano Arithmetic and explain truth by convention in terms of derivability from those principles (see e.g. Tennant 1997).

We also note that our conventionalism allows for a certain degree of pluralism about both logic and mathematics. For reasons of space, we can only offer the briefest sketch of our view, which in outline is this. If there is a plurality of competing conventions for the use of a given logical or mathematical term *e*, then there will be a plurality of theories of the notion expressed by *e*. Consider, for instance, smooth infinitesimal analysis (Bell 2008; Shapiro 2014). This is a perfectly acceptable mathematical theory, albeit one that requires the logic to be intuitionistic, since it explodes if we apply to it exclusively classical principles such as the rule of double negation elimination (DNE). On our view, smooth infinitesimal analysis is true in the (intuitionistic) language in which it’s stated. Likewise, intuitionistic logic is correct, as applied to smooth infinitesimal analysis. In this sense, we are pluralist about logic and mathematics. But our pluralism is broadly Carnapian (see Carnap 1937): rather than taking there to be a plurality of consequence relations for a single language (see e.g. Beall and Restall 2006), we take there to be a plurality of languages each of which has as much claim to correctness as the others.^40^ Smooth infinitesimal analysis and classical analysis are theories stated in different languages—they give different meanings to ‘real number’ and are correctly interpreted by different structures. That is, they are simply *about* different things. Likewise, classical logic and intuitionistic logic give different meanings to, for instance, the negation sign.^41^

## Objections and replies

We begin by considering the worry, alluded to in §2.4.4, that relying on McGee’s Rule amounts to begging the question. This objection, which Field (2001, pp. 355–356) advances in response to McGee’s (1997, pp. 57–61) attempt to secure arithmetical determinacy by appeal to an open-ended interpretation of the first-order induction schema, can be put as follows: McGee’s Rule immediately entails that we can introduce into our language a new predicate that determinately applies to the natural numbers. And though it doesn’t follow that our language *currently* contains any such predicate, it does follow that we’re able, if we wish, to determinately make it the case that the extension of a given predicate is the set of natural numbers. But the worry that gave rise to the MSC in the first place was precisely that naturalism doesn’t seem to leave room for a mechanism by which we can do this. In this context, then, maintaining that we can fix extensions in the way McGee’s Rule says we can requires supposing that some mysterious, possibly supernatural reference-fixing mechanism is, if not currently in use, then at least available for use in the future. So our account of the categoricity of arithmetic fails to be naturalist-friendly in the way we’ve claimed it is—an open-ended account relying on McGee’s Rule can, at best, allow us to ‘carry postulated future magic over to the present; and future magic is no less mysterious than present magic’ (Field 2001, p. 356).

This objection, though, targets an interpretation of McGee’s Rule that’s *not* the interpretation we’re relying on. As we pointed out in §2.4.4, McGee’s Rule, as we’re understanding it, doesn’t say that we’re able to add to our language terms naming any items we like; it’s a minimal thesis about what sorts of languages (understood as abstract objects) exist, not a thesis about what sorts of languages we have a way of coming to speak. And this minimal thesis is enough for our purposes for the simple reason that open-endedness requires not only that our logical rules be valid in any expansion of our language that we’re in fact able to speak—it requires that they be valid in any expansion of our language that’s logically possible. Given that our quantifier rules are open-ended in this sense, all that’s needed to guarantee the truth of Theorems 10 and 12 is for everything to be named in some logically possible expansion of our language. We need not, and do not, suppose that some mysterious reference-fixing mechanism is available.

Of course, there remains the question of whether, if open-endedness requires so much, our quantifier rules are genuinely open-ended. Our explanation of why those rules are open-ended was that we’re disposed to accept all instances of them, irrespective of the situation we find ourselves in—in particular, irrespective of what new terms are in the language we find ourselves speaking. But that explanation can be successful only if the dispositions in question cover even situations that, as a matter of fact, we have no feasible way of coming to be in—for instance, situations in which we speak a *determinately interpreted mathematical language*. Can our dispositions really stretch so far?

We think they can. The reason it’s plausible that we’re disposed to infer in accordance with our logical rules irrespective of what new terms are in our language, recall, is that our dispositions are syntactic. To be disposed to infer in accordance with a given rule is to be disposed to accept a sentence of a particular syntactic form whenever one accepts other sentences of particular syntactic forms. And insofar as a disposition is sensitive only to what the syntactic forms are of the sentences we accept, it’s *not* sensitive to what new terms are in the language in which our logical expressions are embedded. But if that’s right, then, plausibly, our dispositions do indeed cover situations we have no feasible way of getting ourselves into: regardless of whether there is in fact a feasible way for us to come to be in a situation in which our language contains a new term determinately naming some particular item, our psychologies are structured in such a way that we’re disposed, even when faced with a situation in which our language *does* contain such a term, to infer in accordance with our logical rules. Of course, if there’s no feasible way for us to come to be in such a situation, these dispositions will never be *manifested* in such a situation. But that’s not what matters. What matters is just that the dispositions aren’t *sensitive* to whether we’re in such a situation.^42^ (Compare: regardless of whether there’s a feasible way for a given bathroom mirror to come to be in a situation in which it’s on the surface of Mars, the mirror’s molecules are structured in such a way that it’s disposed, even when faced with a situation in which it *is* on the surface of Mars, to shatter on being struck with a hammer. If there’s no feasible way for the mirror to come to be in such a situation, this disposition will never be *manifested* in such a situation. But that’s not what matters. What matters is just that the disposition isn’t *sensitive* to whether the mirror is in such a situation.)

But perhaps this is too quick. The problem here, one might insist, is not just that there are items such that there’s no *feasible* way for us to get ourselves into situations in which our language contains new terms determinately naming those items—it’s that there are items such that there’s not even any *possible* way for us to get ourselves into situations in which our language contains new terms determinately naming those items. And one might insist further that, if this is right, then our account commits us to the existence of dispositions covering literally impossible situations, despite the fact that—at least on standard theories on which *N*’s being disposed to $\varphi$ when condition *C* obtains entails the nonvacuous truth of certain subjunctive conditionals describing what *N* would do were *N* to be in some *C*-situation—there are no such dispositions.^43^ Should we be worried about this?

We think not, for two reasons. The first is that we suspect, along with Jenkins and Nolan (2012), that, contrary to what standard theories of dispositions say, there can indeed be dispositions that cover impossible situations. Whether an agent has a particular disposition of the kind we’re interested in is, in our view, a matter of how the agent’s psychology is structured, where what sort of structure is required can be specified non-modally, and so, as long this structure is such that the relevant disposition isn’t sensitive to whether we’re in some particular situation, the possibility or impossibility of our coming to be in that situation just doesn’t seem relevant. But we won’t argue for that view here. We’ll focus instead on the second reason, which is that the situations in question aren’t impossible in the first place—at least, not in any sense that could plausibly make trouble for the claim that there can be dispositions covering those situations.

Consider that the reason standard theories rule out dispositions covering impossible situations is that the orthodox (Lewis–Stalnaker) treatment of subjunctive conditionals is a *possible worlds* semantics: if it’s impossible for *N* to be in a given situation, then there just aren’t any worlds in which *N* is in that situation, in which case there can be no nonvacuously true subjunctive conditionals describing what *N* would do were *N* to be in that situation. Note, though, that the worlds that are relevant, on this orthodox treatment, are the worlds that are *metaphysically* possible—i.e. possible in some absolute sense. So the only dispositions that are ruled out are dispositions covering *metaphysically* impossible situations. And there’s just no plausibility to the claim that the situations we’re interested in are metaphysically impossible. For instance, insofar as arithmetical realism is true, there are metaphysically possible worlds where we become part of a community that includes angels who are in quasi-perceptual contact with abstracta and who thereby can introduce into our language a predicate the extension of which is the set of natural numbers. Of course, there are weaker notions of impossibility on which such worlds *are* impossible—they are, for instance, nomologically impossible, in our view. But that doesn’t matter—dispositions can indeed cover situations that are impossible in these weaker senses. (This is an obvious implication of standard theories of dispositions, and it’s also highly intuitive. It seems clear, for instance, that our mirror can be disposed to shatter on being struck with a hammer even in a situation in which the hammer was just conjured into existence by a sorcerer.^44^) Given all this, we conclude that our dispositions to reason in accordance with our logical rules can stretch as far as they need to for our argument to go through.

Moving on: it’s also been argued that any commitment to higher-order logic is bound to be epistemologically bankrupt (Wright 1985) and that, in any event, open-ended rules fall prey to Putnam’s just-more-theory manoeuvre (Button and Walsh 2018; Button 2019). We briefly consider these objections in turn.

One might object that, in view of the incompletability of SOL, one can neither acquire nor manifest knowledge of the meanings of the second-order quantifiers: as a matter of mathematical fact, such knowledge wildly exceeds the methods of proof available to us and must therefore be seen as bogus.^45^ However, the objection fails to convince: on our account, speakers *can* learn, and manifest knowledge of, the meanings of $\forall _2$ and $\exists _2$. All this requires is that speakers be disposed to infer according to these quantifiers’ (open-ended) I- and E-rules.

Relatedly, it might be insisted the notion of open-endedness is itself indeterminate, and hence can be of no help in securing the determinacy of our logical and mathematical language. As Button and Walsh put it, ‘our grasp on the idea of [open-endedness] is exactly as precarious as our grasp on *full* second-order quantification’ (2018, p. 163). The problem, they suggest, is that to grasp the open-endedness of a rule is to understand that rule as valid in ‘all possible extensions of our language’, and so an instance of the just-more-theory manoeuvre rears its head: a vicious circularity has been introduced, since ‘to explain what ‘all possible extensions of our language’ amounts to, we must already have grasped the semantic notions which are employed in full second-order logic’ (2018, p. 314).

Our response to this instance of the just-more-theory manoeuvre is a version of the response we outlined in §1: what secures determinacy, on our view, is just that our rules are open-ended, not that we *grasp* their open-endedness. What’s required in order to grasp open-endedness, then, is simply irrelevant—again, all that’s required for the quantifier rules to *be* open-ended is for speakers’ dispositions to reason in accordance with them to be insensitive to the language in which the quantifiers are embedded. But, again, this is something that immediately falls out of the syntactic character of our dispositions to infer according to basic logical rules (see §2.1 and §2.4.4 above). If one is disposed to infer according to a given open-ended rule *R* with premises of the form $\mathfrak {P}_1, \dots , \mathfrak {P}_n$ and a conclusion of the form $\mathfrak {Q}$, then one is disposed to infer according to *R* irrespective of how our language is expanded. The disposition to infer to a conclusion of logical form $\mathfrak {Q}$ is simply activated whenever the speaker is presented with premises of logical form $\mathfrak {P}_1, \dots , \mathfrak {P}_n$.^46^ So, *pace* e.g. Lavine (1994, p. 227) and Button (2019, §2),^47^ speakers (and theorists) need not grasp the complex mathematical concepts involved in the standard semantics for SOL in order for it to be guaranteed that SOL is correctly interpreted by that semantics. What’s required is simply that speakers have the right syntactic dispositions. And though *describing* open-ended dispositions may involve higher-order resources, *having* the dispositions does not.^48^

## Concluding remarks

We’ve offered a naturalistically acceptable account of the (quasi-) determinacy of both logical and mathematical language—one on which the determinacy of the former provides the key for securing the determinacy (or quasi-determinacy) of the latter. We began by offering an essentially new, and naturalistically acceptable, solution to Carnap’s Categoricity Problem for propositional logic, first-order logic, and higher-order logic, showing that our dispositions to infer according to open-ended logical rules determine the full semantics for higher-order logic. On this basis, we then appealed to standard categoricity results to secure the categoricity (or quasi-categoricity) of mathematical theories.

The inferentialist assumptions on which we’ve relied are exceedingly modest—few would deny that the interpretation of our logical and mathematical expressions is determined by the use we make of them, and many accept, further, that our understanding of these expressions is to be explained by reference to our dispositions to infer according to basic rules of use. But these assumptions yield a surprising result: they allow us to demonstrate, *contra* the likes of Skolem, Putnam, and, more recently, Hamkins and Button and Walsh, that the interpretation of our mathematical vocabulary, realistically construed, isn’t vulnerable to determinacy worries.^49^

The resulting view is a broadly Carnapian form of pluralism about logic and mathematics: though logical and mathematical reality are independent of us, *we* determine, via our patterns of use, what our logical and mathematical language is *about*.

## Footnotes

1 For a criticism of non-truth-conditional approaches to semantics, see Williamson (2007, pp. 281ff.).

2 For moderate logical inferentialism, see e.g. Wagner (1981); McGee (2000); Garson (2013); for a corresponding view of mathematical language, see Benacerraf (1985); Shapiro (1991); McGee (1997). For a global version of the view, see e.g. the ‘global descriptivism’ of Lewis (1984).

3 More precisely, Button and Walsh (2018) argue that moderate inferentialism is untenable if one understands it—as we do—as the claim that our use of the language determines the class of structures, in the model theorist’s sense, that interpret our language.

4 For a precursor to applying open-endedness to the interpretation of second-order quantifiers, see Restall (2008).

5 We keep an open mind on what exactly mathematical structures, identified up to isomorphism, are. If pressed, we’d say that they should be conceived of as equivalence classes of isomorphic structures. However, everything we say is compatible with alternative views, such as Stewart Shapiro’s *ante rem* structuralism (Shapiro 1997). We should also point out that although, for simplicity, in the main text we mostly talk about categoricity, everything we say applies to the weaker notion of $\kappa$-categoricity, where, for a given cardinal $\kappa$, a theory is $\kappa$-categorical if it has at least one model of cardinality $\kappa$ and all such models are isomorphic (see e.g. Hodges 1993, §12.5).

6 See e.g. Lavine (1994, 2006); Parsons (1990); McGee (1997, 2000, 2006).

7 For discussion of the former problem, see e.g. Field (2001, pp. 353–354), Warren and Waxman (2020, p. 489), Walmsley (2002, p. 253), and Button and Walsh (2018, pp. 163, 314). For discussion of the latter problem, see Brîncuş (2019) and §2.4.1 below.

8 Incidentally, it also establishes that, if permutation invariance or anything in the neighborhood is *sufficient* for logicality—if, for instance, McGee is right that a connective is logical ‘if and only if it follows from the meaning of the connective that it is invariant under arbitrary bijections’ (1996, p. 578)—then, *pace*Quine (1970), full second-order logic *is* logic.

9 For a classic statement of this broadly Tarskian view, see Field (1972). For a more recent proposal, see Glanzberg (2015).

10 See e.g. Dummett (1973), Putnam (1977, 1980, 1981), Shapiro (1991, §8.3), and Button and Walsh (2018, §7.1). Even an anti-inferentialist such as Williamson (1994, §7.5) accepts that the meaning of a linguistic expression supervenes on its use, and hence counts as a moderate inferentialist in our sense.

11 See e.g. Skolem (1923), Putnam (1977, 1980, 1981), Benacerraf (1985), Wright (1985), Button (2013), and Button and Walsh (2018, §7.3).

12 For a recent presentation, discussion, and development of the argument, see Button (2013) and, especially, Button and Walsh (2018).

13 See e.g. Putnam (1977, pp. 486–487, 494; 1980, p. 477; 1981, pp. 45–48).

14 See e.g. Lewis (1984, pp. 224–225), Hale (2017, p. 715), Bays (2001, pp. 341–348).

15 For a different view, see Button (2013, Ch. 4 and especially Chs. 5–6).

16 The most natural option here—the one we discuss in the main text—is to adopt full second-order logic. But other options are available—e.g. one may try to secure categoricity by appealing to the expressibility of essentially second-order notions such as finitude and computability. However, we don’t think these attempts present any real advantage over the adoption of full second-order logic: just as in the case of the higher-order quantifiers, notions such as finitude and computability can be deviantly interpreted within a first-order setting, and no set of principles can force them to have their intended second-order interpretation (see e.g. Button and Smith 2012; Dean 2014). Still another possible move is to take a page from Lewis, who famously argues that the world itself provides the required determinacy. In his view, naturalness has a role to play in determining reference: natural objects and properties—i.e. those that ‘respect the objective joints in nature’ (1984, p. 227)—act as ‘reference magnets’, so that, when our best theory of a domain is satisfied by many distinct interpretations, the correct interpretation turns out to be the one that maximizes naturalness. (For more discussion of reference magnetism, see Williams (2007), Bays (2007), and Sider (2011).) Whatever the merits of this view as it applies to empirical language (in our view: not many), it is not clear whether it generalises to logical and mathematical language. (For one thing, it’s unclear whether the full interpretation of second-order logic is more ‘natural’ than, say, interpretations on which the second-order quantifiers range over certain definable subsets of the domain. For another, it’s equally unclear which models of ZFC (plus certain large cardinal assumptions) can be most naturally seen as characterising the cumulative hierarchy—at least, this is an issue on which experts still wildly disagree.) One final alternative is to appeal to the $\omega$-rule—closing PA under this rule delivers True Arithmetic, the theory that includes all truths expressible in the language of arithmetic (Carnap 1937; Warren 2020b). There are two problems here, however: (i) the $\omega$-rule is non-recursive—i.e. it’s not possible to tell, even in principle, whether any putative instance of the rule *is* an instance—and (ii) even if the $\omega$-rule were acceptable, appealing to it by itself wouldn’t help us in answering the MSC anyway, since True Arithmetic is itself non-categorical.

17 See e.g. Button and Walsh (2018, Ch. 7).

18 See e.g. Zermelo (1930) and Button and Walsh (2018, §8.3).

19 For general background on SOL and its semantics, see Shapiro (1991, §4.2) and Button and Walsh (2018, Ch. 1).

20 See e.g. Field (2001, p. 352), McGee (2006, pp. 185–186), Button and Walsh (2018, §7.7 and references therein).

21 Proponents of this view include, among many others, Hodes (2004), McGee (2000, 2006), and Garson (2013). For general background on inferentialism, see Murzi and Steinberger (2017) and the references therein. For a recent dispositionalist response to Kripke’s (1982) famous criticisms of dispositional accounts of rule-following, see Warren (2020a).

22 Williamson (2003, 2007) objects to inferentialism by appeal to the claim that speakers who refuse to infer in accordance with the basic rules for a given logical expression may nevertheless be credited with a full understanding of the ordinary meaning of that expression. But, we think, this claim is false. For a sketch of an answer to Williamson’s objections, see Murzi and Steinberger (2017, §4).

23 Multiple-conclusions approaches fail to respect the fact that our practice exclusively involves arguments with at most *one* conclusion (Rumfitt 2008; Steinberger 2011). As for bilateralist approaches, they’re compatible with our inferential practice only on the empirical assumption that the attitude of rejection isn’t to be explained in terms of acceptance but rather is to be understood independently, and it’s not clear that this assumption is supported by the available evidence.

24 Concerning Non-triviality, it may be argued that, since we don’t infer as though all of the sentences of our language are true, the sentences we currently accept must form a consistent set. However, while this would guarantee Non-triviality, the simple fact that we don’t infer as though all of the sentences of our language are true doesn’t by itself show that the interpretation of our language must be consistent. For instance, it may be that, even though our language *is* trivial—say, because our legal system is inconsistent (so that, if our logic is intuitionistic or classical, every sentence is true)—we don’t treat it as such simply for *pragmatic* reasons. As for Compositionality, it may well be that, as is often argued, we’re entitled to it on the grounds that it is ‘our currently best explanation of [a productive language’s] learnability’ (Bonnay and Westerståhl 2016, p. 725). That said, Compositionality has been challenged on various grounds: see e.g. Fodor (2001), Brandom (2008); for an overview of some of the main challenges to the principle of compositionality, see e.g. Pagin and Westerståhl (2010, §2) and Pelletier (2016). For these reasons, we think it’s worth showing that we can solve Carnap’s Problem just by appeal to our inferential dispositions, without relying on either Non-triviality or Compositionality.

25 We note in passing that McGee (2000) also assumes Non-triviality.

26 Peirce’s Law, $((\varphi \rightarrow \psi ) \rightarrow \varphi ) \rightarrow \varphi$, is a case in point: its proof requires classical *reductio* in addition to the rules for $\rightarrow$.

27 We’re also assuming, as usual, that at most *x* is free in $\varphi$ and that any bound variable in $\varphi$ is different from *x*.

28 Both of these refinements, incidentally, are reasonable from an inferentialist perspective. As for the former: there are various things to say here, but one simple point to make is that $\bot$ doesn’t appear to work at all in the way we expect propositional expressions to work. For instance, it’s used only in proofs, where there’s no need to suppose that it expresses anything at all—its only function seems to be to indicate that something has gone wrong, that ‘a story being spun out [has gotten] tied up in a particular kind of knot’ (Tennant 1999, p. 204). And as for the latter: when we assume and discharge sentences, we’re reasoning with what are essentially temporary ‘*ad hoc* axioms’ (Schroeder-Heister 1984, p. 1285) in order to determine something about how derivability works in our system, and it seems obvious that, just as it’s possible for us to reason with *ad hoc* axioms, it’s also possible for us to reason with *ad hoc* rules.

29 That is, Murzi’s system is *separable*. Incidentally, Murzi also shows that the rules of his system are harmonious. Both separability and harmony are properties that standard axiomatisations of CPL lack but that inferentialists often take to be essential to any adequate axiomatisation of logic.

30 Notice that this generalised definition gives the previous one as a special case when all the formulas in the sequent are sentences.

31 Here we are taking for granted that inferentialists can reasonably assume that valuations are *subatomic*—i.e. that the grammatical category of a given expression determines what kind of thing can be the referent of that expression, so that it’s guaranteed that the referents of singular terms are objects, that the referents of one-place predicates are properties (i.e. sets of objects), and so on. Garson (2013, Ch. 17) shows that, if we don’t assume at least this much, we don’t even guarantee that the quantifiers range over a subset of the domain. (Note that this by no means requires that we assume that the expressions in question have their intended referents; it requires only that we assume that their referents are guaranteed to be of a certain *kind*.)

32 Consider, for instance, a sentence $\varphi$ such that neither $\varphi$ nor $\lnot \varphi$ is a theorem of CPL, and let *C* be a class containing just those valuations with respect to which the rules of CPL are valid but in which neither $\varphi$ nor $\lnot \varphi$ is true. We know that *C* has at least one member: Carnap’s provability valuation $v^\dagger$. (Oddly, McGee doesn’t consider this valuation—he doesn’t acknowledge Carnap’s Categoricity Problem at all. His primary concern, in the case of CPL, seems to be ruling out the intuitionistic interpretation of $\lnot$.) We also know that, if any sentence is true in $v^\dagger$, it’s a theorem, in which case it’s true in every valuation with respect to which the rules of CPL are valid. But there are such valuations that aren’t in *C*: valuations that respect the classical meaning of $\lnot$, for instance. So there can be no sentence true in exactly the valuations in *C*, contrary to McGee’s Principle.

33 We only need to worry about $\forall$ because we define $\exists$ as $\lnot \forall \lnot$, in both the first- and the second-order case.

34 We do know that the variables range at least over every object to which some closed term refers. For suppose otherwise. Then, for some closed term *t*, $\forall x \lnot (x = t)$ is true in *v*, and so, by the local validity of $\forall$-E, $\lnot (t = t)$ is true in *v* as well. But this is impossible, since $t = t$ is a theorem. We also know, by similar reasoning, that all the variables have the *same* range. (See also the proof of Theorem 10 below.)

35 To anticipate: our view is that, since our inferential dispositions are syntactic, we are disposed to infer according to the rules for the quantifiers even in circumstances in which we speak determinately interpreted expansions of our language—our dispositions, since they’re sensitive only to syntactic features of our expressions, are *not* sensitive to a language’s interpretation. To be sure, it might be replied that, if the Putnamian skeptic is right, there are *no* circumstances in which we speak such languages. We discuss this reply in §4 below. We’re grateful to an anonymous referee for pressing us to clarify our position here.

36 We note in passing that essentially the same proof can be used to show directly, without any appeal to permutation invariance, that, if $\forall$-E is open-ended, then the Weakened First-Order Thesis entails the First-Order Thesis. (Again we prove the contrapositive. Suppose the Weakened First-Order Thesis doesn’t entail the First-Order Thesis. Then, in some model *v* of FOL, the range of $\forall$ is not the domain itself but some proper subset of the domain, in which case there’s some object not in the range of $\forall$. Let some new term *c* name this object—we know that this is possible, by clause 1 of McGee’s Rule. Then $\forall x \lnot (x = c)$ is true in *v*, and so $\forall$-E can no longer be valid: it would allow us to infer $\lnot (c = c)$, which of course can’t be true in *v*. So $\forall$-E isn’t open-ended.) We go via permutation invariance simply because it ensures that our result is available even to those who don’t share our inferentialist assumptions, so long as they accept the topic neutrality of logic. Thanks to an anonymous referee for pressing us to clarify our position here.

37 Notice that, while the permutation invariance of the interpretation of the first-order quantifier $\forall$ amounts to the invariance of its range under all permutations of the domain *M*, what the permutation invariance of the interpretation of $\forall _2$ requires is somewhat different. Since we are now quantifying over relations rather than objects, the range of $\forall _2$, when it binds a variable of arity *n*, must remain invariant under all permutations, not of *M* itself, but of $\mathcal {P}(M^n)$—i.e. the set of *n*-ary relations on *M*.

38 We’re grateful to Denis Bonnay for helping us appreciate this point.

39 McGee (1997) offers a categoricity result for set theory that doesn’t rely on full SOL. But it is an *internal* categoricity result, in the sense of Walmsley (2002, §4), and so it isn’t a result about the semantics of mathematical vocabulary at all. As Button and Walsh (2018, p. 253) emphasise, ‘it is *literally a type-confusion* to think that an internal result could directly pin down certain [model-theoretic structures]’. What we’re offering, by contrast, is an external—i.e. genuinely semantic—(quasi-) categoricity result. For more on the distinction between external and internal categoricity results, see Button and Walsh (2018, Chs. 10–11).

40 For an argument that Beall and Restall’s pluralism is committed to a version of Carnapian pluralism, see Kouri Kissel (2018).

41 It might be thought that our form of pluralism is under threat from a well-known theorem proved by Harris (1982), which states that any system containing both intuitionistic and classical negation is one in which intuitionistic negation collapses into classical negation. But this objection would be too quick. On our view, intuitionistic and classical negation belong to separate languages, whereas Harris’s theorem only applies to a single language containing both negations.

42 Incidentally, we suspect that McGee had something like this picture in mind all along—this, we take it, is what’s behind his description of ‘psychological and social constraints on what collections we are, in fact, capable of naming’ as ‘beside the point’ (1997, p. 58). So McGee’s argument here, in our view, doesn’t fail by being question-begging. Indeed, we don’t think it fails at all, at least if one isn’t worried about the determinacy of our logical vocabulary and is looking only for a demonstration of arithmetical determinacy in particular. That said, we take our discussion in §§2–3 to show that the best approach to securing (first-order) logical determinacy renders McGee’s argument for arithmetical determinacy unnecessary: our strategy for securing an unrestricted interpretation of the first-order quantifiers extends naturally to the second-order quantifiers as well, in which case Dedekind’s categoricity theorem is available.

43 For discussion of the strengths and weaknesses of standard conditional-based theories of dispositions, see e.g. Manley and Wasserman (2008).

44 Fara (2009, §3) insists that, even when a situation is impossible only in some weak sense, no disposition can cover that situation. But this is highly implausible, for reasons discussed by e.g. Clarke (2009, pp. 334–335) and Vetter and Jaster (2017, pp. 4–5).

45 See e.g. Shapiro (1991, §8.3) and Wright (1985, p. 129). On the learnability and and manifestability requirements, see e.g. Dummett (1973) and Button (2019, §1).

46 To be sure, the disposition can be overridden for all kinds of reasons. For more discussion of this point, see Murzi and Steinberger (2013).

47 See also Shapiro (1991, p. 207).

48 Williamson makes the point in a related context, noting—convincingly, in our view—that because speakers who infer in accordance with open-ended rules ‘may lack the conceptual apparatus necessary to give a reflective account of their own practices’, they need not ‘themselves have a conception of all predicates in all possible extensions of the language’ (Williamson 2006, p. 379).

49 Moreover, this result remains available even independently of our inferentialist assumptions, on the alternative (widely accepted) assumption that permutation invariance is necessary for logicality—in that case, the logicality of the quantifiers is what guarantees the full interpretation of SOL and so makes available the relevant categoricity theorems.
